# Clinical significance of controlling nutritional status score in predicting ICU admission, prolonged hospitalization, and in-hospital mortality in emergency sepsis: a single-center retrospective study

**DOI:** 10.3389/fnut.2026.1888542

**Published:** 2026-09-16

**Authors:** Sue Zhao, Shuang Wu, Yingjie Su, Zengliang Liu, Rong Huang, Zhiwei Yang

**Affiliations:** 1 Department of Respiratory and Critical Care Medicine, The Affiliated Changsha Central Hospital, Hengyang Medical School, University of South China, Changsha, China; 2 Department of Emergency Medicine, The Affiliated Changsha Central Hospital, Hengyang Medical School, University of South China, Changsha, China

**Keywords:** CONUT, emergency department, mortality, prognosis, sepsis

## Abstract

**Background and objective:**

Sepsis is associated with high mortality in critically ill patients. Existing prognostic tools are often complex or require specialized testing. This study aimed to evaluate the prognostic value of the Controlling Nutritional Status (CONUT) score in patients with sepsis in the emergency department.

**Methods:**

A retrospective analysis of 1,200 sepsis patients admitted to the emergency department of the Affiliated Changsha Central Hospital, Hengyang Medical School, University of South China from January 2020 to June 2025 was conducted. Kaplan–Meier analysis, multivariable Cox regression, restricted cubic splines (RCS), receiver operating characteristic (ROC) curves, and decision curve analysis (DCA) were employed to evaluate the associations between the CONUT score and ICU admission, prolonged hospitalization, and in-hospital all-cause mortality. Subgroup and sensitivity analyses were performed.

**Results:**

Time-dependent Cox regression revealed that each one-point increase in the CONUT score was associated with an 11% increase in the risk of in-hospital mortality (HR = 1.11, 95% CI:1.02–1.21). After adjusting for potential confounders, logistic regression analysis further demonstrated that, the CONUT score was significantly associated with ICU admission (OR = 1.16, 95% CI: 1.09–1.23) and prolonged hospitalization (OR = 1.11, 95% CI: 1.05–1.17). RCS revealed linear positive correlations. ROC analysis showed that CONUT was superior to SOFA for predicting prolonged hospitalization, and combining CONUT with SOFA enhanced predictive performance. DCA indicated the combined model provided the highest clinical net benefit. Advanced age, diabetes, and mechanical ventilation modified the associations between CONUT and certain outcomes.

**Conclusion:**

Elevated CONUT scores are independently associated with adverse outcomes in emergency department sepsis patients, serving as a simple prognostic indicator for early risk stratification.

## Introduction

Sepsis represents a critical medical condition wherein an uncontrolled host immune reaction to infection precipitates organ dysfunction, carrying significant morbidity and mortality burdens, and representing a major challenge in emergency and critical care medicine (1, 2). Prompt recognition of individuals at elevated risk and immediate therapeutic intervention are paramount for enhancing patient outcomes. The commonly used clinical prognosis tools include the Acute Physiology and Chronic Health Evaluation II (APACHE II), the quick Sequential Organ Failure Assessment (qSOFA), and the full Sequential Organ Failure Assessment (SOFA). However, these scoring systems exhibit heterogeneous predictive performance across different regions and clinical settings, and some rely on laboratory parameters or complex calculations, limiting their rapid applicability in emergency departments (2–4). Therefore, exploring simple, objective, and readily obtainable prognostic indicators based on routine laboratory tests is of considerable clinical value.

The Controlling Nutritional Status (CONUT) score serves as an objective metric for evaluating nutritional status, derived from three readily available laboratory parameters: serum albumin, total cholesterol, and lymphocyte count. The CONUT score has been validated for predicting mortality in elderly hospitalized patients, acute severe pancreatitis, chronic coronary syndrome, diabetic nephropathy, and maintenance hemodialysis patients (5–9). In critically ill populations, the CONUT score demonstrates multidimensional prognostic value: it is significantly correlated with APACHE II, SOFA, and Charlson Comorbidity Index (all *p* < 0.05), suggesting its capacity to integratively reflect disease severity, and it is significantly associated with intensive care unit (ICU) mortality (10). Multiple investigations in sepsis have revealed significant correlations between the CONUT score and unfavorable clinical outcomes. Specifically, an elevated CONUT score (≥ 4) independently predicts systemic inflammatory response syndrome after percutaneous nephrolithotomy and sepsis onset in medical inpatients (11, 12). In a recent multicenter study from South Korea (Baek et al., *N* = 9,763), the CONUT score was associated with 30-day mortality in a mixed inpatient sepsis population recruited from five hospitals, including ICU, ward, and emergency department settings (13). The prognostic value of the CONUT score in septic patients initially evaluated in the emergency department (ED) remains unclear.

This retrospective study aimed to investigate the associations between the CONUT score and ICU admission, prolonged hospitalization, and in-hospital mortality in ED patients with sepsis, and to evaluate its prognostic value, thereby providing evidence to support early risk stratification, optimization of medical resource allocation, and timely intervention in this population.

## Methods

### Study population

We conducted a retrospective cohort analysis. Pregnant women and patients with COVID-19 infection were excluded. Between January 2020 and June 2025, a total of 3,381 adult patients with suspected sepsis initially presented to the ED. We first excluded 6 patients with prehospital cardiac arrest, 70 with a Sequential Organ Failure Assessment (SOFA) score of 0, 131 with a SOFA score of 1, and 114 with missing SOFA data, leaving 3,060 patients who fulfilled the Sepsis-3 diagnostic criteria. Of these, 198 were not hospitalized (16 died in the ED, and 182 were either kept under ED observation or transferred to other hospitals), yielding 2,862 hospitalized patients. We further excluded 146 patients with missing serum albumin, 54 with missing lymphocyte counts, and 1,462 with missing total cholesterol measurements. Finally, 1,200 patients with sepsis were included in the analysis, of whom 71, 401, 505, and 223 were classified as CONUT grades 1 (normal), 2 (mild), 3 (moderate), and 4 (severe), respectively (Figure 1).

**Figure 1:**
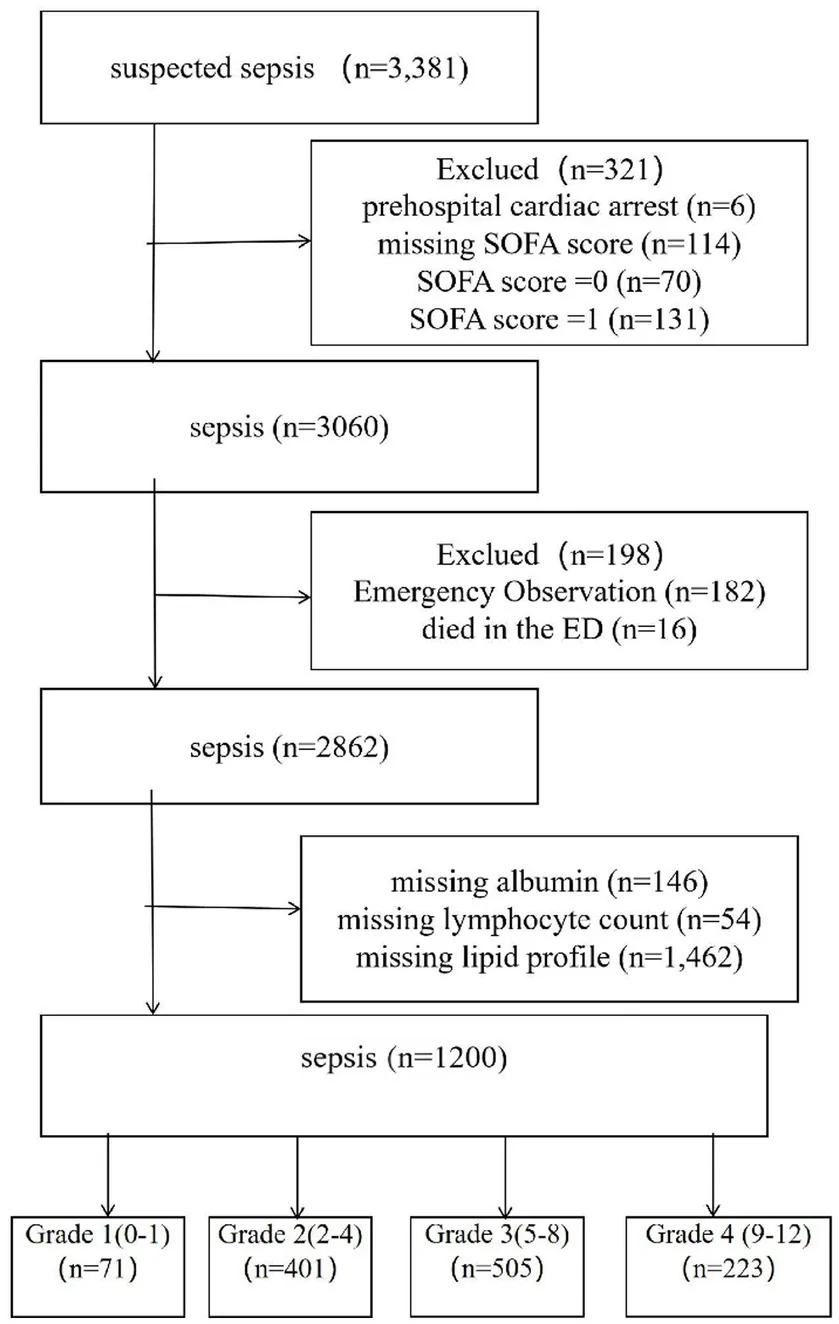
Study flowchart of patient selection and CONUT score stratification.

The study protocol was approved by the Ethics Committee of the Affiliated Changsha Central Hospital, Hengyang Medical School, University of South China (Approval Number: 2025 Medical Review No. 305).

### Evaluation and calculation of CONUT

The CONUT score was computed by aggregating three component scores derived from serum albumin, total cholesterol, and peripheral lymphocyte count, as described in prior literature (11). Study participants were categorized into four nutritional status groups based on cumulative scores: normal nutritional status (0–1 points), mild malnutrition (2–4 points), moderate-to-severe malnutrition (5–8 points), and severe malnutrition (9–12 points). Specific scoring parameters are detailed in Table 1.

**Table 1:** The controlling nutritional status scoring system.

Parameters	Degree			
	Normal	Light	Moderate	Severe
Serum albumin (g/dL)	≥3.5	3.0–3.49	2.50–2.99	<2.50
Score	0	2	4	6
Total lymphocyte count (/mm3)	≥1,600	1,200–1,599	800–1,199	<800
Score	0	1	2	3
Total cholesterol (mg/dL)	≥180	140–179	100–139	<100
Score	0	1	2	3
CONUT score	0–1	2–4	5–8	9–12

CONUT, controlling nutritional status.

### Calculation of the SOFA score

The SOFA score was employed to evaluate organ dysfunction severity across six physiological domains: respiratory, cardiovascular, hepatic, coagulation, neurological, and renal. Each domain received a graded score from 0 to 4, reflecting either physiological derangement magnitude or therapeutic intervention intensity, producing an aggregate range of 0–24. Elevated scores corresponded to progressively worse organ function.

### The definition of sepsis and septic shock

Sepsis and septic shock were defined according to the Third International Consensus Definitions (Sepsis-3): sepsis was defined as infection with an acute increase in SOFA score of ≥2 points, and septic shock as a vasopressor requirement to maintain MAP ≥65 mmHg together with serum lactate >2 mmol/L despite adequate fluid resuscitation (14, 15).

## Data collection

Research data were retrieved from the emergency department’s electronic health record platform. The included covariates refer to previously published related papers (16). Baseline characteristics of enrolled patients included vital signs at emergency department presentation (heart rate, blood pressure), demographic characteristics (sex, age), laboratory findings, medical history, and comorbidities. All data were extracted and verified by researchers who had received standardized training.

Laboratory parameters were defined as the first test results obtained after emergency department presentation. Specifically, lipid profiles were derived from the first blood draw in the emergency department or the first lipid panel after hospital admission. Hematological parameters were measured using the Beckman Coulter LH750 automated hematology analyzer. Biochemical parameters included total cholesterol, alanine aminotransferase (ALT), aspartate aminotransferase (AST), albumin, serum potassium, direct bilirubin, and creatinine, were measured using the Beckman Coulter AU400 analyzer. Arterial blood gas parameters were measured using the Siemens RAPID point 500 blood gas analyzer.

Recorded comorbidities included cerebrovascular disease (17), hypertension (18), coronary heart disease (19), diabetes mellitus (20), heart failure (21), liver disease, and malignant tumor. Liver disease was defined as a documented diagnosis of any of the following: hepatitis, cirrhosis, hepatic abscess, liver rupture with bleeding, or portal hypertension. Malignant tumor was defined as a current diagnosis of malignancy at any anatomical site. Additionally, the following variables were collected: emergency department triage area, site of infection, mechanical ventilation and surgical treatment status, total hospital length of stay, emergency department length of stay (EDLOS), and medical expenses.

### Study follow-up and primary endpoints

The study endpoints included ICU admission, prolonged hospitalization, and in-hospital all-cause mortality. Prolonged hospitalization was defined as a total hospital length of stay exceeding 14 days, which corresponded to the upper quartile (75th percentile) of the distribution of hospitalization duration (22). Clinical outcomes were obtained through review of medical records.

### Statistical analysis

Continuous variables with normal distribution were presented as mean ± standard deviation, while those with non-normal distribution were expressed as median with interquartile range. Categorical variables were summarized as frequencies and percentages. Comparative analyses across groups employed parametric one-way analysis of variance for normally distributed continuous data, non-parametric Kruskal-Wallis testing for skewed continuous distributions, and chi-square test for categorical variables.

We constructed sequentially adjusted Cox proportional hazards regression models to evaluate the association between the CONUT score and clinical outcomes in ED patients with sepsis. For the outcome of in-hospital mortality, important predictors were selected from 22 candidate variables using least absolute shrinkage and selection operator (LASSO) regression (Supplementary Figures 1, 2 and Supplementary Table 1). SHAP (SHapley Additive exPlanations) analysis was used to interpret the contribution of each variable to the model predictions. Based on SHAP importance and prediction analyses (Supplementary Figures 3, 4) and previous literature, seven covariates were ultimately included for adjustment: sex, age, mechanical ventilation, EDLOS, diabetes, cerebrovascular disease, and SOFA score. Multicollinearity among these covariates was assessed using the variance inflation factor (VIF); all VIF values were < 5, indicating no substantial collinearity (Supplementary Table 2). The proportional hazards (PH) assumption was examined for each of the seven covariates with respect to in-hospital mortality using the Schoenfeld residuals test. SOFA violated the PH assumption, whereas all other covariates satisfied it (Supplementary Table 3). Accordingly, in-hospital mortality was analyzed using a time-dependent Cox regression model. For external time-dependent covariates, we constructed interaction terms between the baseline covariate (X) and a time function f(t). The model was specified as:

h(t|X)=h�(t)exp[αX+βX·f(t)]

Where h₀(t) denotes the baseline hazard function, α represents the time-invariant effect of X, and β quantifies the time-dependent modification of the effect. The natural logarithm of follow-up time, f(t) = ln(t), was selected as the time function. The results are presented in Table 2. Survival probabilities across CONUT categories were estimated using the Kaplan–Meier method and compared with the log-rank test.

**Table 2:** The association of controlling nutritional status (CONUT) score with clinical outcomes (in-hospital all-cause mortality) in 1200 emergency department sepsis patients.

Exposure	Model I		Model II		Model III		Model IV	
	HR (95%CI)	*P*	HR (95%CI)	*P*	HR (95%CI)	*P*	HR (95%CI)	*P*
In-hospital all-cause mortality								
CONUT	1.15 (1.06 ~ 1.24)	**<0.001**	1.15 (1.06 ~ 1.24)	**<0.001**	1.10(1.02 ~ 1.20)	**0.017**	1.11(1.02 ~ 1.21)	**0.016**
SOFA	**—**	**—**	**—**	**—**	1.55 (1.36 ~ 1.78)	**<0.001**	1.56 (1.36 ~ 1.78)	**<0.001**
Time-dependent coefficients of SOFA	**—**	**—**	**—**	**—**	0.441–0.101 × ln(t)		0.442–0.101 × ln(t)	
CONUT		**<0.001**		**0.001**		**0.020**		**0.018**
CONUT0-1	1.00 (Reference)		1.00 (Reference)		1.00 (Reference)		1.00 (Reference)	
CONUT2-4	2.53 (0.33 ~ 19.18)	0.368	2.30(0.30 ~ 17.50)	0.419	2.09 (0.28 ~ 15.94)	0.474	2.15 (0.29 ~ 16.35)	0.461
CONUT5-8	3.41 (0.46 ~ 25.02)	0.228	3.03 (0.41 ~ 22.31)	0.276	2.36 (0.32 ~ 17.49)	0.399	2.49(0.33 ~ 18.67)	0.374
CONUT9-12	7.65 (1.04 ~ 56.16)	**0.045**	6.72(0.91 ~ 49.49)	0.061	4.64 (0.62 ~ 34.47)	0.134	4.92 (0.65 ~ 37.16)	0.122
SOFA	**—**	**—**	**—**	**—**	1.55 (1.35 ~ 1.78)	**<0.001**	1.55(1.35 ~ 1.78)	**<0.001**
Time-dependent coefficients of SOFA	**—**	**—**	**—**	**—**	0.436–0.101 × ln(t)		0.436–0.101 × ln(t)	

Model I adjust for: none. Model II adjust for: gender, Age. Model III adjust for: gender, age, ventilation, EDLOS, diabetes, cerebrovascular disease, time-dependent SOFA variables. Model IV adjust for: gender, age, ventilation, EDLOS, diabetes, cerebrovascular disease, time-dependent SOFA variables, NLR. EDLOS, Length of stay in the emergency department; CONUT, controlling nutritional status; SOFA, the Sequential Organ Failure Assessment; NLR, Neutrophil to Lymphocyte Ratio; HR, Hazard Ratio; CI, Confidence Interval.

The risks of ICU admission and prolonged hospitalization were similarly analyzed. Important predictors were again selected from the 22 candidate variables using LASSO regression (Supplementary Figures 5, 6), and SHAP importance and prediction analyses were performed (Supplementary Figures 7, 8). Eighteen covariates were ultimately included for adjustment: sex, age, mechanical ventilation, triage department, mean arterial pressure (MAP), EDLOS, hypertension, coronary heart disease, diabetes, cerebrovascular disease, SOFA score, liver disease, malignancy, hemoglobin, neutrophil-to-lymphocyte ratio (NLR), platelet count, creatinine, and potassium (Supplementary Table 4). Multicollinearity among the 18 covariates was assessed using the VIF; all values were < 5, indicating no substantial collinearity (Supplementary Table 5). Sequentially adjusted logistic regression models were then constructed for ICU admission and prolonged hospitalization.

Restricted cubic splines (RCS) were applied to investigate potential dose–response relationships between CONUT scores with ICU admission and prolonged hospitalization. Predictive discrimination was assessed through receiver operating characteristic (ROC) curve analysis with area under the curve calculation (AUC). Model performance was further compared using the DeLong test for AUCs, and the incremental predictive value of adding CONUT to SOFA was quantified by categorical and continuous net reclassification improvement (NRI) and integrated discrimination improvement (IDI). Decision curve analysis (DCA) evaluated clinical utility. To verify the robustness of our findings, we further conducted sensitivity analyses using multiple imputation for missing variables, together with subgroup analyses for the outcomes of ICU admission and prolonged hospitalization.

All statistical tests were two-tailed with significance set at *p* < 0.05. Analyses were performed using SPSS26.0, R version 4.3.3, and Zstats 1.0 software platforms.

## Results

### Patient characteristics

The study comprised 1,200 enrolled participants. The mean age was 74.87 ± 13.13 years, 69.2% were male, and the predominant site of infection was the lung (77.8%). According to the CONUT score, patients were stratified into four groups: 0–1 points (*n* = 71, 5.9%), 2–4 points (*n* = 401, 33.4%), 5–8 points (*n* = 505, 42.1%), and 9–12 points (*n* = 223, 18.6%). Marked variations in numerous clinical parameters were evident across the groups (Table 3).

**Table 3:** Baseline characteristics of 1,200 study participants.

Characteristics	Total (*N* = 1,200)	CONUT0-1 (*N* = 71)	CONUT2-4 (*N* = 401)	CONUT5-8 (*N* = 505)	CONUT9-12 (*N* = 223)	*p*-value
Age, years (Mean ± SD)	74.87 ± 13.13	70.24 ± 12.61	75.40 ± 11.77	75.05 ± 13.85	74.97 ± 13.70	0.022
Age stratification, *n* (%)						0.121
<65 years	233 (19.42%)	19 (26.76%)	68 (16.96%)	95 (18.81%)	51 (22.87%)	
≥65 years	967 (80.58%)	52 (73.24%)	333 (83.04%)	410 (81.19%)	172 (77.13%)	
Gender, *n* (%)						0.063
Female	369 (30.8%)	28 (39.4%)	106 (26.4%)	159 (31.5%)	76 (34.1%)	
Male	831 (69.2%)	43 (60.6%)	295 (73.6%)	346 (68.5%)	147 (65.9%)	
Hypertension, *n* (%)						0.115
No	567 (47.2%)	29 (40.8%)	180 (44.9%)	238 (47.1%)	120 (53.8%)	
Yes	633 (52.8%)	42 (59.2%)	221 (55.1%)	267 (52.9%)	103 (46.2%)	
Coronary heart disease, *n* (%)						0.098
No	818 (68.2%)	47 (66.2%)	262 (65.3%)	342 (67.7%)	167 (74.9%)	
Yes	382 (31.8%)	24 (33.8%)	139 (34.7%)	163 (32.3%)	56 (25.1%)	
Diabetes, *n* (%)						0.086
No	837 (69.8%)	49 (69.0%)	296 (73.8%)	349 (69.1%)	143 (64.1%)	
Yes	363 (30.2%)	22 (31.0%)	105 (26.2%)	156 (30.9%)	80 (35.9%)	
Cerebrovascular disease, *n* (%)						<0.001
No	837 (69.8%)	51 (71.8%)	303 (75.6%)	349 (69.1%)	134 (60.1%)	
Yes	363 (30.2%)	20 (28.2%)	98 (24.4%)	156 (30.9%)	89 (39.9%)	
Heart failure, *n* (%)						0.460
No	1,096 (91.3%)	64 (90.1%)	370 (92.3%)	464 (91.9%)	198 (88.8%)	
Yes	104 (8.7%)	7 (9.9%)	31 (7.7%)	41 (8.1%)	25 (11.2%)	
Liver disease, *n* (%)						0.086
No	1,174 (97.83)	70 (98.59)	397 (99.00)	493 (97.62)	214 (95.96)	
Yes	26 (2.17)	1 (1.41)	4 (1.00)	12 (2.38)	9 (4.04)	
Malignant tumor, *n* (%)						0.009
No	1,101 (91.75)	68 (95.77)	379 (94.51)	459 (90.89)	195 (87.44)	
Yes	99 (8.25)	3 (4.23)	22 (5.49)	46 (9.11)	28 (12.56)	
Respiratory failure, *n* (%)						<0.001
No	748 (62.3%)	31 (43.7%)	230 (57.4%)	326 (64.6%)	161 (72.2%)	
Yes	452 (37.7%)	40 (56.3%)	171 (42.6%)	179 (35.4%)	62 (27.8%)	
SOFA score (Median [Q1, Q3])	3.00(2.00,5.00)	3.00(2.00,3.00)	3.00(2.00,4.00)	4.00(3.00,5.00)	4.00(3.00,7.00)	<0.001
SOFA score groups, *n* (%)						<0.001
2–4	821 (68.42)	64 (90.14)	315 (78.55)	330 (65.35)	112 (50.22)	
≥ 5	379 (31.58)	7 (9.86)	86 (21.45)	175 (34.65)	111 (49.78)	
Systolic blood pressure, mmHg (Median[Q1, Q3])	130.00 (110.00, 150.00)	148.00 (122.50,168.75)	136.00 (121.00,150.00)	127.00 (108.00,146.50)	120.00 (99.00,139.00)	<0.001
Diastolic blood pressure, mmHg (Median[Q1, Q3])	73.00(60.00,85.00)	86.00(73.00,98.50)	76.00(65.00,88.00)	72.00(57.00,83.00)	67.00(55.00,76.00)	<0.001
Mean arterial pressure, mmHg (Median[Q1, Q3])	91.00 (78.00, 105.00)	108.00 (89.00,122.50)	96.00 (84.00,108.00)	90.00 (74.00,104.00)	84.00 (70.00,94.00)	<0.001
White blood cells, 109/L (Median[Q1, Q3])	9.98(7.03,14.35)	10.19(8.45,15.26)	10.44(7.33,14.00)	9.66(6.43,13.69)	10.21(6.56,16.52)	0.025
Hemoglobin, g/L (Median[Q1, Q3])	120.50(103.00,135.00)	134.00(125.00,147.00)	130.00(116.00,142.00)	117.00(100.00,131.00)	101.00(80.50,117.50)	<0.001
Neutrophil cells, 109/L (Median[Q1, Q3])	8.30(5.39,12.43)	6.60(5.52,10.04)	8.39(5.39,12.29)	8.30(5.26,12.16)	8.94(5.63,14.68)	0.062
Lymphocyte cells, 109/L (Median[Q1, Q3])	0.79(0.47,1.20)	2.10(1.78,3.06)	1.05(0.72,1.46)	0.61(0.40,0.95)	0.60(0.36,0.84)	<0.001
Neutrophil to lymphocyte ratio	10.61 (5.37, 20.67)	3.22 (2.04,4.31)	8.01 (4.44,14.56)	13.19 (7.24,23.27)	16.61 (8.18,28.10)	<0.001
Platelets, 109/L (Median[Q1, Q3])	174.00(125.75,247.00)	235.00(154.50,275.00)	187.00(141.00,249.00)	159.00(118.00,230.00)	147.00(88.00,252.00)	<0.001
Albumin,g/L (Median[Q1, Q3])	34.00(29.00,38.00)	41.00(38.50,43.00)	37.00(35.00,40.00)	32.00(30.00,35.00)	25.00(23.00,27.00)	<0.001
Alanine aminotransferase, U/L(Median[Q1, Q3])	20.00(12.00,36.00)	20.00(14.50,31.50)	19.00(12.00,31.00)	20.00(12.00,37.00)	22.00(11.50,43.50)	0.648
Aspartate aminotransferase, U/L (Median[Q1, Q3])	27.00(19.00,45.25)	24.00(19.00,35.00)	25.00(19.00,37.00)	28.00(19.00,47.00)	31.00(20.00,61.00)	<0.001
Direct bilirubin, umol/L (Median[Q1, Q3])	4.10(2.59,8.07)	2.60(1.74,4.05)	3.60(2.40,5.74)	4.80(2.80,8.84)	5.36(3.00,12.34)	<0.001
Creatinine, umol/L (Median[Q1, Q3])	97.00(68.00,158.00)	75.00(59.50,104.00)	86.00(66.00,124.00)	102.00(72.00,170.00)	129.00(74.50,223.50)	<0.001
Cholesterol, mmol/L (Median[Q1, Q3])	3.31(2.63,4.05)	4.76(4.18,5.60)	3.96(3.29,4.50)	3.10(2.55,3.60)	2.38(1.94,2.92)	<0.001
Potassium, mmol (Median[Q1, Q3])	4.00(3.60,4.40)	3.90(3.70,4.30)	4.00(3.60,4.30)	3.90(3.50,4.50)	4.00(3.50,4.60)	0.830
Ventilation, *n* (%)						0.075
No	1,038 (86.5%)	56 (78.9%)	354 (88.3%)	442 (87.5%)	186 (83.4%)	
Yes	162 (13.5%)	15 (21.1%)	47 (11.7%)	63 (12.5%)	37 (16.6%)	
Operation, *n* (%)						<0.001
No	1,174 (97.8%)	71 (100%)	399 (99.5%)	494 (97.8%)	210 (94.2%)	
Yes	26 (2.2%)	0 (0%)	2 (0.5%)	11 (2.2%)	13 (5.8%)	
Infection sites, *n* (%)						<0.001
Urinary tract	103 (8.6%)	2 (2.8%)	35 (8.7%)	48 (9.5%)	18 (8.1%)	
Abdomen	101 (8.4%)	1 (1.4%)	15 (3.7%)	44 (8.7%)	41 (18.4%)	
Lungs	933 (77.8%)	64 (90.1%)	335 (83.5%)	387 (76.6%)	147 (65.9%)	
Other	63 (5.2%)	4 (5.6%)	16 (4.0%)	26 (5.1%)	17 (7.6%)	
Triage department, *n* (%)						0.088
Emergency internal medicine or emergency surgery	174 (14.5%)	6 (8.5%)	60 (15.0%)	84 (16.6%)	24 (10.8%)	
Resuscitation room	1,026 (85.5%)	65 (91.5%)	341 (85.0%)	421 (83.4%)	199 (89.2%)	
Length of stay in the emergency department, *n* (%)						0.825
<4 h	508 (42.3%)	28 (39.4%)	167 (41.6%)	213 (42.2%)	100 (44.8%)	
≥4 h	692 (57.7%)	43 (60.6%)	234 (58.4%)	292 (57.8%)	123 (55.2%)	
Management, days (Mean ± SD)	13.02 ± 12.74	11.11 ± 7.40	11.63 ± 10.46	13.85 ± 14.51	14.22 ± 13.28	0.015
Expenses, dollars (Median[Q1, Q3])	2823.12(1732.73,5971.49)	2083.41(1459.22,3640.34)	1988.33(1414.47,3500.79)	3052.87(1962.56,6477.54)	5276.09(3183.00,10009.70)	<0.001
Admission to the intensive care unit, *n* (%)						<0.001
No	770 (64.2%)	54 (76.1%)	312 (77.8%)	321 (63.6%)	83 (37.2%)	
Yes	430 (35.8%)	17 (23.9%)	89 (22.2%)	184 (36.4%)	140 (62.8%)	
Prolonged hospital stay, *n* (%)						<0.001
≤14 days	880 (73.3%)	58 (81.7%)	325 (81.0%)	348 (68.9%)	149 (66.8%)	
>14 days	320 (26.7%)	13 (18.3%)	76 (19.0%)	157 (31.1%)	74 (33.2%)	
In-hospital all-cause mortality, *n* (%)						<0.001
No	1,123 (93.6%)	70 (98.6%)	386 (96.3%)	475 (94.1%)	192 (86.1%)	
Yes	77 (6.4%)	1 (1.4%)	15 (3.7%)	30 (5.9%)	31 (13.9%)	

CONUT, controlling nutritional status; SOFA, the Sequential Organ Failure Assessment.

Individuals with elevated CONUT scores tended to be of advanced age, had higher NLR, and demonstrated greater prevalence of cerebrovascular disease, heart failure, and malignant tumor. Regarding laboratory findings, hemoglobin, lymphocyte count, platelet count, albumin, and total cholesterol levels decreased significantly with increasing CONUT scores. Conversely, AST, direct bilirubin, and serum creatinine levels increased significantly as the CONUT score rose. CONUT score categories were significantly and positively correlated with the SOFA score (*p* < 0.001). Furthermore, the rates of ICU admission, medical costs, prolonged hospitalization, and in-hospital all-cause mortality increased significantly with higher CONUT scores (all *p* < 0.001).

Kaplan–Meier survival analysis revealed a stepwise relationship between CONUT score strata and mortality risk among septic patients (Figure 2).

**Figure 2:**
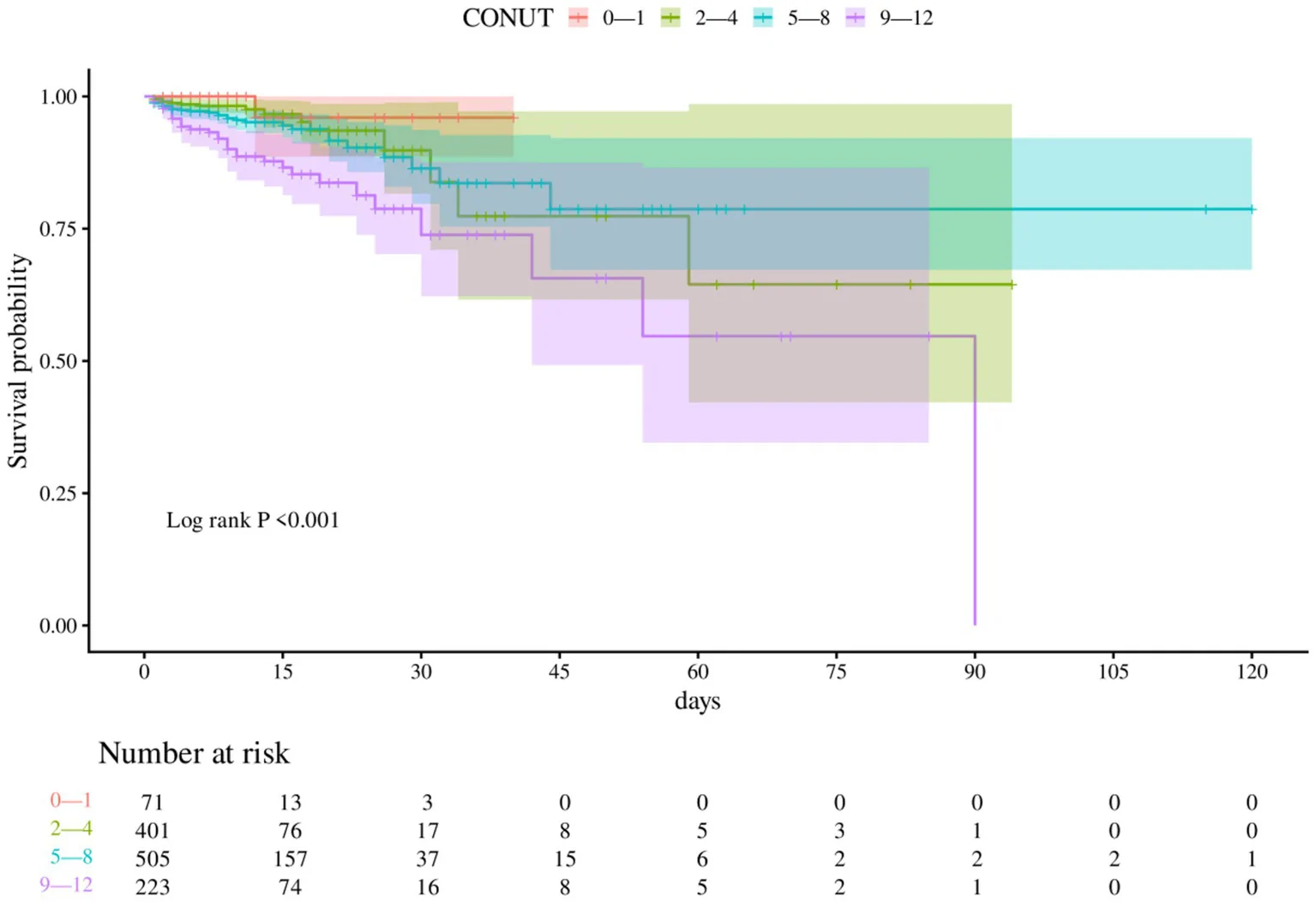
Kaplan–Meier survival analyses were used to assess the associations between CONUT score and in-hospital all-cause mortality in emergency department sepsis patients. CONUT, controlling nutritional status.

### CONUT score and in-hospital all-cause mortality

As shown in Table 2, when analyzed as a continuous variable, the CONUT score was independently associated with in-hospital all-cause mortality. Each one-point increase in CONUT was associated with a 15% higher risk of in-hospital death in the unadjusted model (HR 1.15, 95% CI 1.06–1.24, *p* < 0.001), and the association remained significant after full adjustment for demographics, ventilation, EDLOS, comorbidities, time-dependent SOFA, and NLR (Model IV: HR 1.11, 95% CI 1.02–1.21, *p* = 0.016). SOFA violated the proportional hazards assumption and was therefore modeled with a time-dependent coefficient [0.442–0.101 × ln(t)], indicating that its prognostic effect attenuated over time. When classified by CONUT, trend tests were significant in all models (*p* = 0.018, model IV), although after multivariable adjustment, each category lost statistical significance, possibly due to the limited number of events after CONUT grouping.

CONUT score and ICU admission: In both the crude and adjusted models, the CONUT score was significantly and positively associated with ICU admission in patients with sepsis. In the fully adjusted model(Model III), every point increase in CONUT raised the risk of ICU admission by 16% (OR 1.16, 95% CI 1.09–1.23, *p* < 0.001). Higher CONUT scores corresponded to an increased risk of ICU admission. Trend tests across the three models were statistically significant (*p* < 0.001) (Table 4).

**Table 4:** The association of controlling nutritional status (CONUT) score with clinical outcomes (admission to the intensive care unit and prolonged hospitalization >14 days) in 1200 emergency department sepsis patients.

Exposure	Model I			Model II			Model III	
	OR (95%CI)	*P*		OR (95%CI)	*P*		OR (95%CI)	*P*
Admission to the intensive care unit								
CONUT	1.23 (1.18 ~ 1.29)	**<0.001**		1.23 (1.18 ~ 1.29)	**<0.001**		1.16 (1.09 ~ 1.23)	**<0.001**
CONUT								
CONUT0-1	1.00 (Reference)			1.00 (Reference)			1.00 (Reference)	
CONUT2-4	0.91 (0.50 ~ 1.64)	0.745		0.96 (0.53 ~ 1.75)	0.898		0.93 (0.44 ~ 1.94)	0.840
CONUT5-8	1.82 (1.03 ~ 3.23)	**0.041**		1.92 (1.08 ~ 3.42)	**0.027**		1.44 (0.68 ~ 3.05)	0.341
CONUT9-12	5.36 (2.91 ~ 9.85)	**<0.001**		5.64 (3.06 ~ 10.41)	**<0.001**		3.46 (1.53 ~ 7.81)	**0.003**
P for trend	1.26 (1.21 ~ 1.33)	**<0.001**		1.26 (1.21 ~ 1.33)	**<0.001**		1.19 (1.12 ~ 1.27)	**<0.001**
Prolonged hospitalization> 14 days								
CONUT	1.13 (1.08 ~ 1.18)	**<0.001**		1.13 (1.08 ~ 1.18)	**<0.001**		1.11 (1.05 ~ 1.17)	**<0.001**
CONUT								
CONUT0-1	1.00 (Reference)			1.00 (Reference)			1.00 (Reference)	
CONUT2-4	1.04 (0.54 ~ 2.00)	0.898		1.04 (0.54 ~ 1.99)	0.917		1.17 (0.59 ~ 2.30)	0.650
CONUT5-8	2.01 (1.07 ~ 3.78)	**0.030**		2.01 (1.07 ~ 3.77)	**0.031**		2.04 (1.03 ~ 4.04)	**0.040**
CONUT9-12	2.22 (1.14 ~ 4.30)	**0.019**		2.21 (1.14 ~ 4.30)	**0.019**		1.92 (0.91 ~ 4.05)	0.089
P for trend	1.11 (1.06 ~ 1.17)	**<0.001**		1.11 (1.06 ~ 1.17)	**<0.001**		1.08 (1.01 ~ 1.14)	**0.014**

Model I adjust for: none. Model II adjust for: gender, age. Model III adjust for: gender, age, ventilation, triage department, MAP, EDLOS, hypertension, coronary heart disease, diabetes, cerebrovascular disease, SOFA, liver disease, malignant tumor, hemoglobin, NLR, platelets, creatinine, potassium. MAP, Mean arterial pressure; EDLOS, Length of stay in the emergency department; CONUT, controlling nutritional status; SOFA, the Sequential Organ Failure Assessment; NLR, Neutrophil to Lymphocyte Ratio; OR, Odds Ratio; CI, Confidence Interval.

CONUT score and prolonged hospitalization: In the unadjusted analysis, each one-point increment in CONUT score corresponded to a 13% elevation in prolonged hospitalization risk (OR = 1.13, 95%CI: 1.08–1.18, *p* < 0.001). Following adjustment for gender and age (Model II), this relationship persisted (OR = 1.13, 95% CI: 1.08–1.18, *p* < 0.001). After comprehensive multivariable adjustment for all potential confounders (Model III), the CONUT score maintained its independent associated with prolonged hospitalization (OR = 1.11, 95% CI: 1.05–1.17, *p* = 0.001). Trend tests achieved statistical significance across all three analytical models (Table 4).

### Restricted cubic spline analysis

Following comprehensive adjustment for all potential confounders, restricted cubic spline analysis revealed linear positive relationships between the CONUT score with prolonged hospitalization (> 14 days) and ICU admission (Figures 3A,B).

**Figure 3:**
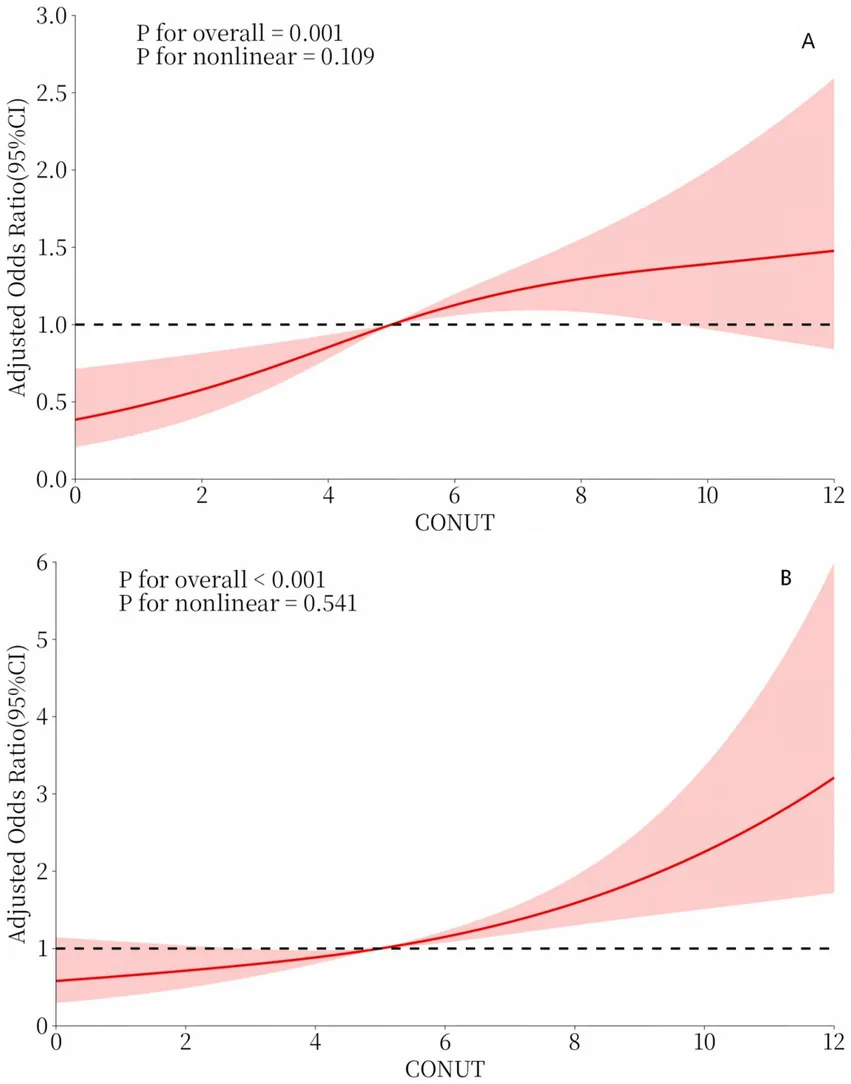
Logistic regression using restricted cubic spline regression of CONUT score with prolonged hospitalization **(A)** and ICU admission **(B)**. Adjusted for gender, age, ventilation, triage department, MAP, EDLOS, hypertension, coronary heart disease, diabetes, cerebrovascular disease, SOFA, liver disease, malignant tumor, hemoglobin, NLR, platelets, creatinine, potassium. MAP, Mean arterial pressure; EDLOS, Length of stay in the emergency department; CONUT, controlling nutritional status; SOFA, he Sequential Organ Failure Assessment; NLR, Neutrophil to Lymphocyte Ratio.

### Predictive performance evaluation

The discriminatory ability of CONUT and SOFA scores was assessed through receiver operating characteristic curve analysis (Figures 4A–C). Regarding ICU admission prediction, the CONUT score yielded an AUC of 0.667 (95% CI: 0.634–0.699), inferior to the SOFA score’s discriminatory capacity (AUC: 0.736; 95%CI: 0.706–0.765). Their combination enhanced predictive performance to 0.759 (95%CI: 0.730–0.787). For prolonged hospitalization, the CONUT score demonstrated superior discrimination (AUC: 0.606; 95% CI: 0.571–0.642) compared to SOFA (AUC: 0.557; 95% CI: 0.521–0.593), with modest improvement in the combined model (AUC: 0.612; 95% CI: 0.577–0.648). In in-hospital all-cause mortality prediction, CONUT achieved an AUC of 0.637 (95%CI: 0.557–0.718), again below SOFA’s performance (AUC: 0.738; 95%CI: 0.654–0.822). The integrated CONUT-SOFA model attained the highest discriminatory value of 0.762 (95%CI: 0.685–0.838). AUCs and model comparisons are provided in Supplementary Tables 6, 7, respectively.

**Figure 4:**
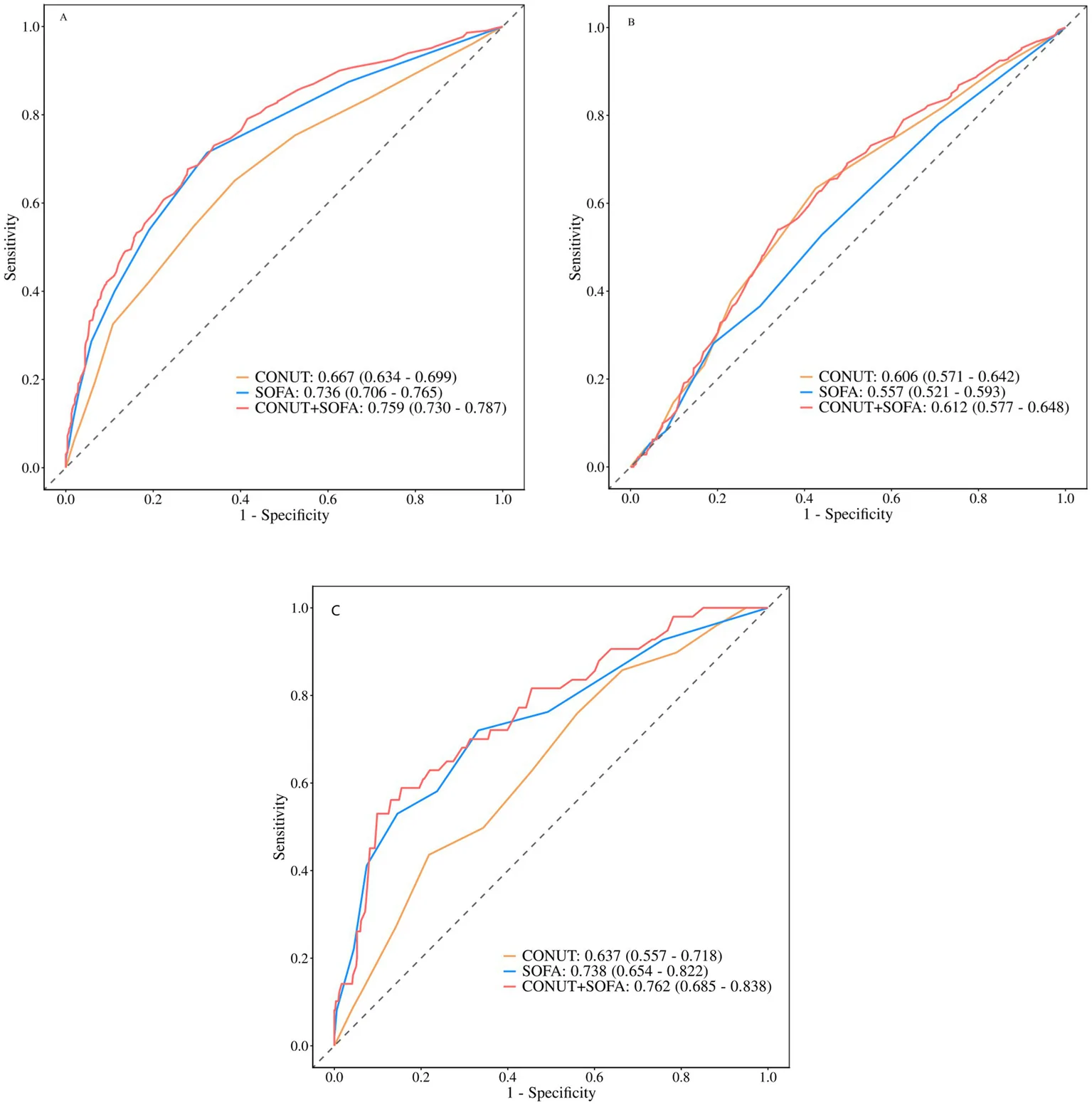
Receiver operating characteristic curves assesses the predictive capability of the CONUT score for ICU admission **(A)**, prolonged hospitalization **(B)**, and in-hospital all-cause mortality **(C)**. CONUT, controlling nutritional status; SOFA, the Sequential Organ Failure Assessment; ICU, intensive care unit.

Compared with SOFA alone, the combined CONUT + SOFA model significantly improved risk reclassification and discrimination for ICU admission (categorical NRI 0.097, continuous NRI 0.434, IDI 0.032, all *p* < 0.001) and prolonged hospital stay >14 days (categorical NRI 0.093, continuous NRI 0.388, IDI 0.019, all *p* < 0.001), with significant AUC improvements confirmed by DeLong tests (both *p* < 0.01). For in-hospital all-cause mortality, however, adding CONUT to SOFA yielded no statistically significant improvement in NRI or IDI (all *p* > 0.05), although the point estimates were consistently positive (Supplementary Table 7).

### Clinical utility assessment

Decision curve analysis revealed that for predicting ICU admission in septic patients presenting to the emergency department, the integrated CONUT-SOFA model offered superior net benefit across a wide spectrum of risk thresholds, approximately spanning 0 to 86%. For predicting prolonged hospitalization, DCA demonstrated that the combined CONUT and SOFA model provided the highest net benefit within the low-to-moderate risk threshold range (0–34%). However, DCA showed that the combined CONUT–SOFA model did not increase the net benefit compared with the SOFA model alone for predicting 10-day in-hospital mortality (Figures 5A–C and Supplementary Table 8).

**Figure 5:**
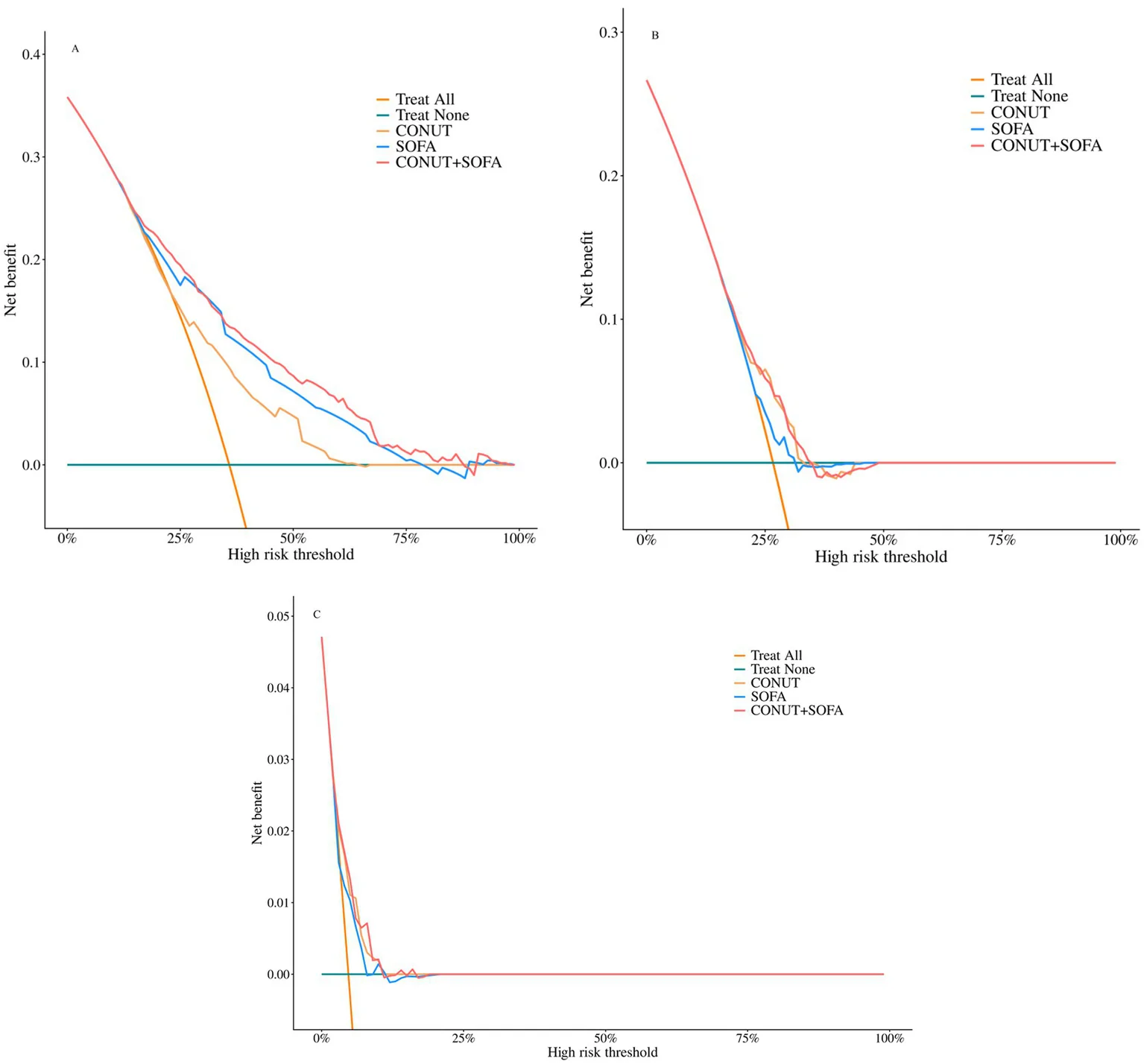
Decision curve analysis for the outcome of ICU admission **(A)**, prolonged hospitalization **(B)**, and in-hospital all-cause mortality **(C)**. EDLOS, Length of stay in the emergency department; CONUT, controlling nutritional status; SOFA, the Sequential Organ Failure Assessment; ICU, intensive care unit.

### Sensitivity analyses

First, missing values of serum albumin, lymphocyte count, and total cholesterol were handled by multiple imputation, and the analyses were repeated in the full cohort of 2,862 hospitalized patients; the results were not materially altered (in-hospital mortality, Table 5; multi-model analyses of prolonged hospital stay and ICU admission, Table 6). Second, the SOFA score was categorized into two groups (Group 1: 2–4; Group 2: ≥5) (23), and standard Cox regression was performed in the 1,200 patients with sepsis; the findings remained consistent (Tables 7, 8). Third, the definition of prolonged hospital stay was varied. When defined as hospitalization ≥10 days, the results were essentially unchanged. When defined as hospitalization ≥21 days, the association remained statistically significant in Models I and II but lost significance in Model III (Table 9), which may be attributable to the reduced number of events and limited statistical power under this stricter definition.

**Table 5:** The association of controlling nutritional status (CONUT) score with clinical outcomes (in-hospital all-cause mortality) in 2862 emergency department sepsis patients.

Exposure	Model I		Model II		Model III		Model IV	
	HR (95%CI)	*P*	HR (95%CI)	*P*	HR (95%CI)	*P*	HR (95%CI)	*P*
In-hospital all-cause mortality								
CONUT	1.14 (1.08 ~ 1.20)	**<0.001**	1.14 (1.08 ~ 1.20)	**<0.001**	1.09(1.02 ~ 1.15)	**0.003**	1.09(1.03 ~ 1.15)	**0.005**
SOFA	**—**	**—**	**—**	**—**	1.46 (1.33 ~ 1.60)	**<0.001**	1.46 (1.33 ~ 1.60)	**<0.001**
Time-dependent coefficients of SOFA	**—**	**—**	**—**	**—**	0.378–0.080 × ln(t)		0.379–0.080 × ln(t)	
CONUT		**<0.001**		**<0.001**		**0.020**		**0.018**
CONUT0-1	1.00 (Reference)		1.00 (Reference)		1.00 (Reference)		1.00 (Reference)	
CONUT2-4	2.39 (0.57 ~ 10.03)	0.233	2.22(0.53 ~ 9.32)	0.275	2.29 (0.55 ~ 9.63)	0.257	2.28 (0.54 ~ 9.58)	0.260
CONUT5-8	3.53 (0.86 ~ 14.43)	0.079	3.21 (0.79 ~ 13.14)	0.105	2.82 (0.69 ~ 11.58)	0.149	2.79(0.68 ~ 11.51)	0.156
CONUT9-12	6.06 (1.48 ~ 24.84)	**0.012**	5.67(1.38 ~ 23.23)	**0.016**	4.36(1.06 ~ 17.98)	**0.041**	4.31 (1.03 ~ 17.92)	**0.045**
SOFA	**—**	**—**	**—**	**—**	1.45(1.33 ~ 1.59)	**<0.001**	1.45(1.33 ~ 1.60)	**<0.001**
Time-dependent coefficients of SOFA	**—**	**—**	**—**	**—**	0.376–0.080 × ln(t)		0.377–0.080 × ln(t)	

Model I adjust for: none. Model II adjust for: gender, Age. Model III adjust for: gender, age, ventilation, EDLOS, diabetes, cerebrovascular disease, time-dependent SOFA variables. Model IV adjust for: gender, age, ventilation, EDLOS, diabetes, cerebrovascular disease, time-dependent SOFA variables, NLR. EDLOS, Length of stay in the emergency department; CONUT, controlling nutritional status; SOFA, the Sequential Organ Failure Assessment; NLR, Neutrophil to Lymphocyte Ratio; HR, Hazard Ratio; CI, Confidence Interval.

**Table 6:** The association of controlling nutritional status (CONUT) score with clinical outcomes (admission to the intensive care unit and prolonged hospital stay > 14 days) in 2862 emergency department sepsis patients.

Exposure	Model I			Model II			Model III	
	OR (95%CI)	*P*		OR (95%CI)	*P*		OR (95%CI)	*P*
Admission to the intensive care unit								
CONUT	1.24 (1.20 ~ 1.27)	**<0.001**		1.24 (1.20 ~ 1.27)	**<0.001**		1.16 (1.12 ~ 1.21)	**<0.001**
CONUT								
CONUT0-1	1.00 (Reference)			1.00 (Reference)			1.00 (Reference)	
CONUT2-4	0.94 (0.63 ~ 1.42)	0.784		0.96 (0.64 ~ 1.45)	0.847		0.84 (0.52 ~ 1.38)	0.494
CONUT5-8	1.91 (1.29 ~ 2.84)	**0.001**		1.93 (1.30 ~ 2.87)	**0.001**		1.32 (0.81 ~ 2.16)	0.271
CONUT9-12	4.74 (3.14 ~ 7.16)	**<0.001**		4.78 (3.16 ~ 7.23)	**<0.001**		2.62 (1.55 ~ 4.44)	**<0.001**
P for trend	1.24 (1.20 ~ 1.28)	**<0.001**		1.24 (1.20 ~ 1.28)	**<0.001**		1.16 (1.12 ~ 1.21)	**<0.001**
Prolonged hospital stay>14 days								
CONUT	1.11 (1.08 ~ 1.14)	**<0.001**		1.11 (1.09 ~ 1.15)	**<0.001**		1.09 (1.05 ~ 1.13)	**<0.001**
CONUT								
CONUT0-1	1.00 (Reference)			1.00 (Reference)			1.00 (Reference)	
CONUT2-4	0.98 (0.67 ~ 1.45)	0.925		0.95 (0.65 ~ 1.41)	0.807		0.96 (0.65 ~ 1.43)	0.851
CONUT5-8	1.45 (0.99 ~ 2.11)	0.054		1.42 (0.97 ~ 2.08)	0.069		1.29 (0.86 ~ 1.92)	0.215
CONUT9-12	2.03 (1.37 ~ 3.01)	**<0.001**		2.01 (1.35 ~ 2.98)	**<0.001**		1.59 (1.02 ~ 2.46)	**0.039**
P for trend	1.10 (1.07 ~ 1.14)	**<0.001**		1.10 (1.07 ~ 1.14)	**<0.001**		1.07 (1.03 ~ 1.11)	**<0.001**

Model I adjust for: none. Model II adjust for: gender, age. Model III adjust for: gender, age, ventilation, triage department, MAP, EDLOS, hypertension, coronary heart disease, diabetes, cerebrovascular disease, SOFA, liver disease, malignant tumor, hemoglobin, NLR, platelets, creatinine, potassium. MAP, Mean arterial pressure; EDLOS, Length of stay in the emergency department; CONUT, controlling nutritional status; SOFA, the Sequential Organ Failure Assessment; NLR, Neutrophil to Lymphocyte Ratio. OR: Odds Ratio, CI: Confidence Interval.

**Table 7:** The association of controlling nutritional status (CONUT) score with clinical outcomes (in-hospital all-cause mortality) in 1200 emergency department sepsis patients.

Variables	Model1			Model2			Model3			Model4	
	HR (95%CI)	*P*		HR (95%CI)	*P*		HR (95%CI)	*P*		HR (95%CI)	*P*
CONUT	1.15 (1.06 ~ 1.24)	**<0.001**		1.15 (1.06 ~ 1.24)	**<0.001**		1.11 (1.02 ~ 1.20)	**0.018**		1.11 (1.02 ~ 1.22)	**0.015**
CONUT											
CONUT0-1	1.00 (Reference)			1.00 (Reference)			1.00 (Reference)			1.00 (Reference)	
CONUT2-4	2.53 (0.33 ~ 19.18)	0.368		2.30 (0.30 ~ 17.50)	0.419		1.82 (0.24 ~ 13.87)	0.565		1.97 (0.26 ~ 15.03)	0.515
CONUT5-8	3.41 (0.46 ~ 25.02)	0.228		3.03 (0.41 ~ 22.31)	0.276		2.26 (0.30 ~ 16.82)	0.426		2.57 (0.34 ~ 19.33)	0.359
CONUT9-12	7.65 (1.04 ~ 56.16)	**0.045**		6.72 (0.91 ~ 49.49)	0.061		4.44 (0.59 ~ 33.30)	0.147		5.08 (0.67 ~ 38.76)	0.117
P for trend	1.19 (1.10 ~ 1.29)	**<0.001**		1.18 (1.09 ~ 1.29)	**<0.001**		1.15 (1.06 ~ 1.25)	**0.001**		1.16 (1.06 ~ 1.27)	**0.001**

Model I adjust for: none. Model II adjust for: gender, Age. Model III adjust for: gender, age, ventilation, EDLOS, diabetes, cerebrovascular disease, SOFA score groups. Model IV adjust for: gender, age, ventilation, EDLOS, diabetes, cerebrovascular disease, SOFA score groups, NLR. EDLOS, Length of stay in the emergency department; CONUT, controlling nutritional status; SOFA, the Sequential Organ Failure Assessment; NLR, Neutrophil to Lymphocyte Ratio; HR, Hazard Ratio; CI, Confidence Interval.

**Table 8:** The association of controlling nutritional status (CONUT) score with clinical outcomes (admission to the intensive care unit and prolonged hospital stay >14 days) in 1200 emergency department sepsis patients.

Exposure	Model I			Model II			Model III	
	OR (95%CI)	*P*		OR (95%CI)	*P*		OR (95%CI)	*P*
Admission to the intensive care unit								
CONUT	1.23 (1.18 ~ 1.29)	**<0.001**		1.23 (1.18 ~ 1.29)	**<0.001**		1.16 (1.09 ~ 1.23)	**<0.001**
CONUT								
CONUT0-1	1.00 (Reference)			1.00 (Reference)			1.00 (Reference)	
CONUT2-4	0.91 (0.50 ~ 1.64)	0.745		0.96 (0.53 ~ 1.75)	0.898		0.91 (0.44 ~ 1.88)	0.794
CONUT5-8	1.82 (1.03 ~ 3.23)	**0.041**		1.92 (1.08 ~ 3.42)	**0.027**		1.44 (0.69 ~ 3.02)	0.331
CONUT9-12	5.36 (2.91 ~ 9.85)	**<0.001**		5.64 (3.06 ~ 10.41)	**<0.001**		3.48 (1.56 ~ 7.75)	**0.002**
P for trend	1.26 (1.21 ~ 1.33)	**<0.001**		1.26 (1.21 ~ 1.33)	**<0.001**		1.20 (1.12 ~ 1.28)	**<0.001**
Prolonged hospital stay> 14 days								
CONUT	1.13 (1.08 ~ 1.18)	**<0.001**		1.13 (1.08 ~ 1.18)	**<0.001**		1.11 (1.05 ~ 1.17)	**<0.001**
CONUT								
CONUT0-1	1.00 (Reference)			1.00 (Reference)			1.00 (Reference)	
CONUT2-4	1.04 (0.54 ~ 2.00)	0.898		1.04 (0.54 ~ 1.99)	0.917		1.17 (0.59 ~ 2.31)	0.648
CONUT5-8	2.01 (1.07 ~ 3.78)	**0.030**		2.01 (1.07 ~ 3.77)	**0.031**		2.05 (1.04 ~ 4.05)	**0.039**
CONUT9-12	2.22 (1.14 ~ 4.30)	**0.019**		2.21 (1.14 ~ 4.30)	**0.019**		1.93 (0.91 ~ 4.08)	0.086
P for trend	1.11 (1.06 ~ 1.17)	**<0.001**		1.11 (1.06 ~ 1.17)	**<0.001**		1.08 (1.02 ~ 1.14)	**0.014**

Model I adjust for: none. Model II adjust for: gender, age. Model III adjust for: gender, age, ventilation, triage department, MAP, EDLOS, hypertension, coronary heart disease, diabetes, cerebrovascular disease, SOFA score groups, liver disease, malignant tumor, hemoglobin, NLR, platelets, creatinine, potassium. MAP, Mean arterial pressure; EDLOS, Length of stay in the emergency department; CONUT, Controlling nutritional status; SOFA score groups, the Sequential Organ Failure Assessment; NLR, Neutrophil to Lymphocyte Ratio; OR, Odds Ratio; CI, Confidence Interval.

**Table 9:** The association of controlling nutritional status (CONUT) score with clinical outcomes (hospital stay ≥10 days and hospital stay ≥21 days) in 1200 emergency department sepsis patients.

Exposure	Model I			Model II			Model III	
	OR (95%CI)	*P*		OR (95%CI)	*P*		OR (95%CI)	*P*
hospital stay ≥10 days								
CONUT	1.09 (1.05 ~ 1.14)	**<0.001**		1.09 (1.05 ~ 1.14)	**<0.001**		1.10 (1.05 ~ 1.16)	**<0.001**
CONUT								
CONUT0-1	1.00 (Reference)			1.00 (Reference)			1.00 (Reference)	
CONUT2-4	1.04 (0.63 ~ 1.73)	0.868		1.07 (0.64 ~ 1.78)	0.808		1.10 (0.65 ~ 1.87)	0.710
CONUT5-8	1.62 (0.98 ~ 2.67)	0.059		1.67 (1.01 ~ 2.76)	**0.047**		1.68 (0.98 ~ 2.89)	0.059
CONUT9-12	1.67 (0.98 ~ 2.86)	0.061		1.68 (0.98 ~ 2.89)	0.060		1.60 (0.87 ~ 2.95)	0.132
P for trend	1.07 (1.03 ~ 1.12)	**<0.001**		1.07 (1.03 ~ 1.12)	**0.001**		1.06 (1.01 ~ 1.12)	**0.024**
hospital stay ≥21 days								
CONUT	1.11 (1.04 ~ 1.17)	**<0.001**		1.10 (1.04 ~ 1.17)	**<0.001**		1.06 (0.99 ~ 1.14)	0.088
CONUT								
CONUT0-1	1.00 (Reference)			1.00 (Reference)			1.00 (Reference)	
CONUT2-4	0.90 (0.40 ~ 2.00)	0.791		0.91 (0.40 ~ 2.05)	0.819		0.94 (0.41 ~ 2.18)	0.891
CONUT5-8	1.40 (0.64 ~ 3.03)	0.400		1.44 (0.66 ~ 3.15)	0.358		1.25 (0.54 ~ 2.90)	0.600
CONUT9-12	1.72 (0.76 ~ 3.87)	0.190		1.73 (0.76 ~ 3.91)	0.190		1.22 (0.48 ~ 3.07)	0.674
P for trend	1.09 (1.03 ~ 1.16)	**0.006**		1.09 (1.02 ~ 1.16)	**0.006**		1.03 (0.96 ~ 1.11)	0.390

Model I adjust for: none. Model II adjust for: gender, age. Model III adjust for: gender, age, ventilation, triage department, MAP, EDLOS, hypertension, coronary heart disease, diabetes, cerebrovascular disease, SOFA score groups, Liver, Tumor, hemoglobin, NLR, Platelets, creatinine, potassium. MAP, Mean arterial pressure; EDLOS, Length of stay in the emergency department; CONUT, controlling nutritional status; SOFA score groups, the Sequential Organ Failure Assessment; NLR, Neutrophil to Lymphocyte Ratio; OR, Odds Ratio; CI, Confidence Interval.

### Subgroup analysis and interaction tests

Stratified analyses and interaction tests demonstrated that the association between the CONUT score and ICU admission remained generally robust across subgroups, although significant interactions were observed in subgroups defined by advanced age and diabetes mellitus (P for interaction < 0.05) (Figure 6A). The association between the CONUT score and prolonged hospitalization was also generally robust across subgroups, with a significant interaction detected in the ventilation and diabetes mellitus subgroups (P for interaction <0.05) (Figure 6B).

**Figure 6:**
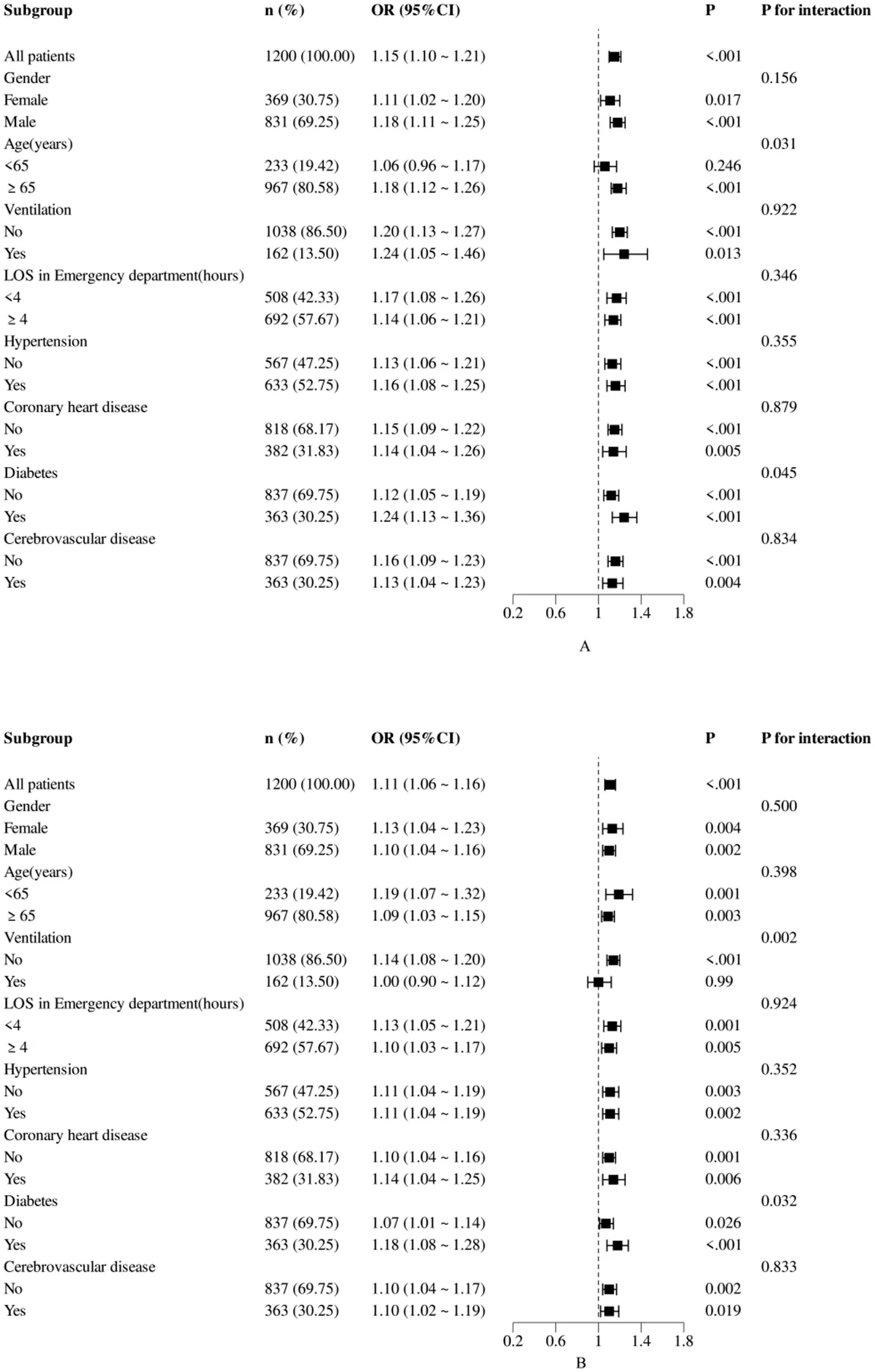
Subgroup analyses of the association between CONUT score and clinical outcomes. **(A)** ICU admission; **(B)** Prolonged hospitalization. CONUT, controlling nutritional status; ICU, intensive care unit; OR, odds ratio; CI, confidence interval; LOS, length of stay.

## Discussion

This study uses the Sepsis-3.0 criteria to diagnose sepsis (16, 24, 25). Our analysis of 1,200 patients demonstrated significant positive associations between elevated CONUT scores and all three adverse outcomes, indicating that heightened CONUT scores serve as an independent predictor of unfavorable clinical events in this population. Subgroup and interaction analyses confirmed the overall robustness of these associations, while revealing that advanced age (≥ 65), ventilation, and diabetes mellitus significantly modified the associations between the CONUT score and both ICU admission and prolonged hospitalization (P for interaction < 0.05). This finding is consistent with previous studies identifying advanced age and diabetes as important risk factors for ICU admission and increased disease burden in sepsis patients (24, 26, 27), mechanical ventilation prolonging ICU stays for sepsis (28). This suggests that nutritional intervention strategies need to be individualized: Older age, diabetes, or mechanical ventilation may heighten susceptibility to adverse clinical outcome through reduced metabolic reserve, compromised immunity, impaired nutrient absorption, and accelerated catabolism.

Standard COX regression and time-dependent COX regression analysis in this study established the CONUT score as an independent predictor of ICU admission, extended hospitalization, and in-hospital all-cause mortality among septic patients. Following comprehensive adjustment for confounding variables, each incremental point in the CONUT score corresponded to a 11% increase in mortality risk (HR = 1.11, 95% CI: 1.02–1.21). Prior research has reported that a high CONUT score is independently associated with prolonged ICU stay (≥ 11.6 days) in patients with severe pneumonia requiring invasive mechanical ventilation (OR = 1.298) (29), which aligns with our finding that each one-point increase in the CONUT score corresponds to a 11% increased risk of prolonged hospitalization (OR = 1.11). Sensitivity analyses using multiple imputation for missing variables yielded consistent conclusions. ROC curve analysis further revealed the clinical application potential of the CONUT score: although the AUC of the CONUT score alone for predicting ICU admission and in-hospital mortality was lower than that of the SOFA score, the combined model significantly improved predictive performance. Notably, the CONUT score demonstrated superior predictive efficacy for prolonged hospitalization (AUC = 0.606) compared with the SOFA score (AUC = 0.557), suggesting its unique clinical value in medical resource planning and early rehabilitation interventions. Integrating the CONUT score into existing clinical assessment systems may enhance risk stratification efficacy and clinical net benefit, further underscoring that nutritional status represents an indispensable dimension in sepsis prognostic evaluation.

These findings align closely with prior cohort studies demonstrating significant associations between elevated CONUT scores and adverse outcomes including in-hospital mortality and prolonged hospitalization. However, the study population in Miano’s research was medical inpatients (11). Using the MIMIC-IV database (*N* = 6,234), Pan et al. reported that the PNI, which is calculated from albumin and lymphocyte count, was associated with short- and medium-term mortality in critically ill patients with sepsis (16). Notably, that study did not incorporate cholesterol, whereas the CONUT score additionally captures energy reserve and lipid metabolism—dimensions that the PNI lacks. In a multicenter cohort of 9,763 patients with sepsis from five hospitals in South Korea, Baek et al. demonstrated that both nutritional indices predicted 30-day mortality (13). Building on these observations, our study offers several distinct contributions. First, unlike previous studies conducted in ICU-based or mixed inpatient populations, our study was performed in the emergency department, where rapid and routinely available laboratory tools are particularly valuable for early triage; all three components of the CONUT score are obtainable immediately upon ED arrival. Second, beyond mortality, we quantified the incremental value of CONUT over SOFA using reclassification metrics and demonstrated significant improvements in NRI and IDI for ICU admission and prolonged hospital stay, thereby extending the role of nutritional assessment to ED disposition decisions. However, adding CONUT to SOFA did not significantly improve NRI or IDI for in-hospital mortality, likely reflecting the strong baseline performance of SOFA, limited death events, or partial overlap between CONUT components and sepsis-induced organ dysfunction.

The CONUT score integrates three parameters—serum albumin (reflecting protein reserves and inflammatory consumption), lymphocyte count (indicating cellular immune function), and total cholesterol (associated with metabolic homeostasis and inflammatory regulation)—providing a comprehensive quantitative assessment tool for the nutritional-immune-inflammatory status in patients with sepsis and SIRS (30, 31). Its value extends beyond the identification of malnutrition to revealing the profound impact of the “nutrition-inflammation-immunity” interplay on clinical outcomes (13, 32, 33). As a key component of the CONUT score, albumin demonstrates a significant positive correlation with sepsis severity: lower serum albumin levels correspond to higher SOFA scores, indicating more severe organ dysfunction and facilitating early identification of high-risk patients (34). Regarding the immune dimension, the absolute peripheral blood lymphocyte count and T-cell subsets are significantly lower in sepsis patients compared with healthy controls, with a progressive decline as the disease advances, suggesting an immunosuppressive state (35, 36). In the metabolic dimension, hypocholesterolemia (<120 mg/dL) is commonly observed in sepsis patients and is closely associated with adverse outcomes; cholesterol metabolic dysregulation participates in the pathophysiological processes of sepsis, influencing immune function and inflammatory response modulation (37, 38). Dynamic changes in cholesterol levels may reflect abnormalities in synthesis, transport, and metabolism induced by sepsis, and its monitoring may assist in assessing disease progression (38). In summary, integrating the CONUT score and the dynamic changes of its individual components into clinical practice may provide a feasible pathway for early identification of high-risk patients and help to take individualized intervention strategies in the emergency setting.

This study has several strengths. Firstly, the study cohort comprised patients with sepsis who were first evaluated in the emergency department. Secondly, the CONUT score is calculated from routinely available laboratory parameters at emergency department presentation, offering computational simplicity and enabling clinicians to rapidly identify patients at high risk of mortality, thereby facilitating timely initiation of early nutritional support and other interventions. Thirdly, this study employed LASSO regression to identify important predictive factors. Covariates were then determined based on SHAP importance and prediction values. Collinearity among the selected covariates was further assessed using VIFs to avoid the model being overfitted. This study has several limitations. First, it was conducted at a single center, making it susceptible to selection and information bias, thereby limiting the external validity and generalizability of our findings. Second, as a retrospective study, the use of vasopressors was not documented, which may have influenced the results. Third, some patients could not be contacted by telephone after discharge; therefore, we were unable to confirm their survival status, and 28-day all-cause mortality analysis was not performed. Furthermore, several factors that may influence sepsis prognosis, such as symptom onset-to-presentation interval, time-to-first antibiotic administration, microbiological results, and potentially other unmeasured confounders could not be retrieved due to the constraints of retrospective data collection.

Subsequent multicenter prospective investigations are imperative to corroborate the prognostic utility of the CONUT score and ascertain whether tailored nutritional strategies guided by CONUT stratification can meaningfully diminish mortality and healthcare resource utilization among septic patients initially evaluated in emergency settings.

## Conclusion

Our findings demonstrated that the CONUT score was positively associated with ICU admission and prolonged hospitalization among sepsis patients presenting to the emergency department, and served as an independent predictor of in-hospital mortality.

## Data Availability

The datasets presented in this article are not readily available because data from this study is available upon reasonable request. Requests to access the datasets should be directed to Zhiwei Yang, 2018050530@usc.edu.cn.

## Supplementary material

SUPPLEMENTARY FIGURE 1 LASSO coefficient profiles of the 22 features in-hospital mortality model.

SUPPLEMENTARY FIGURE 2 Optimal parameter (lambda) selection in the LASSO model used fivefold cross-validation via minimum criteria in-hospital mortality model.

SUPPLEMENTARY FIGURE 3 SHAP (Shapley additive explanations) analysis was used to interpret the contribution of each variable to the in-hospital mortality.

SUPPLEMENTARY FIGURE 4 SHAP analysis was used to interpret the contribution of each variable to the in-hospital mortality.

SUPPLEMENTARY FIGURE 5 LASSO coefficient profiles of the 22 features in both ICU admission and prolonged hospitalization models.

SUPPLEMENTARY FIGURE 6 Optimal parameter (lambda) selection in the LASSO model used fivefold cross-validation via minimum criteria in both ICU admission and prolonged hospitalization models.

SUPPLEMENTARY FIGURE 7 SHAP analysis was used to interpret the contribution of each variable to both ICU admission and prolonged hospitalization models.

SUPPLEMENTARY FIGURE 8 SHAP analysis was used to interpret the contribution of each variable to both ICU admission and prolonged hospitalization models.

SUPPLEMENTARY TABLE 1 LASSO coefficient of the 22 features in-hospital mortality model.

SUPPLEMENTARY TABLE 2 VIF in-hospital mortality model.c

SUPPLEMENTARY TABLE 3 Schoenfeld residuals test in-hospital mortality model.

SUPPLEMENTARY TABLE 4 LASSO coefficient profiles of the 22 features in both ICU admission and prolonged hospitalization models.

SUPPLEMENTARY TABLE 5 Collinearity between ICU admission and prolonged hospitalization: VIF.

SUPPLEMENTARY TABLE 6 The AUC value of the ROC curve.

SUPPLEMENTARY TABLE 7 Clinical reclassification metrics.

SUPPLEMENTARY TABLE 8 The threshold value and net benefit.
