# Supplementary material for: Clinical significance of controlling nutritional status score in predicting ICU admission, prolonged hospitalization, and in-hospital mortality in emergency sepsis: a single-center retrospective study
Source: Front Nutr. 2026 Sep 16;13:1888542. doi: 10.3389/fnut.2026.1888542 (PMC13623573; doi:10.3389/fnut.2026.1888542)
Supplement: Supplementary file 1 [file Table_1.DOCX]

Supplementary table 1

Variables	beta

1.Age	0.013509217

2.SOFA	0.231781185

3.Hemoglobin	0.000000000

4.Platelets	0.000000000

5.AST	0.000443291

6.DBIL	0.000000000

7.Creatinine	0.000000000

8.Potassium	0.000000000

9.CONUT	0.061494685

10.MAP	0.000000000

11.NLR	0.000000000

12.Gender	0.000000000

13.Ventilation	0.190783169

14.Operation	0.000000000

15.EDLOS1	0.107371313

16.Triage department 0.148075489

17.Hypertension1	-0.152769557

18.CHD	0.000000000

19.Diabetes	-0.277081833

20.Cerebrovascular 0.308238667

21.Liver	0.103119177

22.Tumor	0.370704951
