# Supplementary material for: Clinical significance of controlling nutritional status score in predicting ICU admission, prolonged hospitalization, and in-hospital mortality in emergency sepsis: a single-center retrospective study
Source: Front Nutr. 2026 Sep 16;13:1888542. doi: 10.3389/fnut.2026.1888542 (PMC13623573; doi:10.3389/fnut.2026.1888542)
Supplement: Supplementary file 2 [file Table_2.DOCX]

Supplementary table 2:VIF

| Variables | Tolerance | VIF |
| --- | --- | --- |
| Gender | .996 | 1.004 |
| Age | .906 | 1.104 |
| Ventilation | .947 | 1.056 |
| EDLOS | .986 | 1.014 |
| Cerebrovascular | .890 | 1.124 |
| SOFA | .877 | 1.141 |
| CONUT | .907 | 1.103 |
| Diabetes | .981 | 1.019 |
