# Supplementary material for: Clinical significance of controlling nutritional status score in predicting ICU admission, prolonged hospitalization, and in-hospital mortality in emergency sepsis: a single-center retrospective study
Source: Front Nutr. 2026 Sep 16;13:1888542. doi: 10.3389/fnut.2026.1888542 (PMC13623573; doi:10.3389/fnut.2026.1888542)
Supplement: Supplementary file 3 [file Table_3.DOCX]

Supplementary Table 3

| partial residual of variable | | Rankofmanagement1 |
| --- | --- | --- |
| Gender | Pearson correlation | .068 |
|  | Sig. (2-tailed) | .556 |
| Age | Pearson correlation | .133 |
|  | Sig. (2-tailed) | .250 |
| Ventilation | Pearson correlation | -.159 |
|  | Sig. (2-tailed) | .167 |
| EDLOS | Pearson correlation | .185 |
|  | Sig. (2-tailed) | .108 |
| SOFA | Pearson correlation | -.360^**^ |
|  | Sig. (2-tailed) | .001 |
| Diabetes | Pearson correlation | -.179 |
|  | Sig. (2-tailed) | .120 |
| Cerebrovascular | Pearson correlation | .114 |
|  | Sig. (2-tailed) | .323 |
| CONUT | Pearson correlation | -.129 |
|  | Sig. (2-tailed) | .264 |
| Rankoftime | Pearson correlation | 1 |
|  | Sig. (2-tailed) |  |
