# Supplementary material for: Clinical significance of controlling nutritional status score in predicting ICU admission, prolonged hospitalization, and in-hospital mortality in emergency sepsis: a single-center retrospective study
Source: Front Nutr. 2026 Sep 16;13:1888542. doi: 10.3389/fnut.2026.1888542 (PMC13623573; doi:10.3389/fnut.2026.1888542)
Supplement: Supplementary file 4 [file Table_4.DOCX]

Supplementary Table 4

Variables	beta

1.Age	-0.01079723618

2.SOFA	0.32032897570

3.Hemoglobin	-0.00269995939

4.Platelets	0.00090163051

5.AST	0.00000000000

6.DBIL	0.01650378612

7.Creatinine	-0.00001121169

8.Potassium	0.05855254732

9.CONUT	0.13578010648

10.MAP	-0.00916892130

11.NLR	0.00646930370

12.Gender	-0.17605748345

13.Ventilation	2.48254188963

14.Operation	-0.17997398473

15.EDLOS	-0.53648625125

16.Triage department 0.38867846523

17.Hypertension	0.00000000000

18.CHD	-0.00633900475

19.Diabetes	0.00000000000

20.Cerebrovascular	0.16179983366

21.Liver	0.00000000000

22.Tumor	0.24705117273
