# Supplementary material for: Clinical significance of controlling nutritional status score in predicting ICU admission, prolonged hospitalization, and in-hospital mortality in emergency sepsis: a single-center retrospective study
Source: Front Nutr. 2026 Sep 16;13:1888542. doi: 10.3389/fnut.2026.1888542 (PMC13623573; doi:10.3389/fnut.2026.1888542)
Supplement: Supplementary file 5 [file Table_5.DOCX]

ICU和住院延长协变量共线性VIF

| Variables | VIF |
| --- | --- |
| Gender | 1.070 |
| Age | 1.309 |
| Ventilation | 1.150 |
| MAP | 1.262 |
| EDLOS | 1.046 |
| Triage department | 1.066 |
| Hypertension | 1,267 |
| Cerebrovascular | 1.153 |
| Diabetes | 1.095 |
| CHD | 1.148 |
| Liver | 1.022 |
| Tumor | 1,032 |
| SOFA | 1.511 |
| CONUT | 1.585 |
| Hemoglobin | 1.509 |
| NLR | 1.168 |
| Platelets | 1.157 |
| creatinine | 1.148 |
| Potassium | 1.104 |
