# Supplementary material for: Clinical significance of controlling nutritional status score in predicting ICU admission, prolonged hospitalization, and in-hospital mortality in emergency sepsis: a single-center retrospective study
Source: Front Nutr. 2026 Sep 16;13:1888542. doi: 10.3389/fnut.2026.1888542 (PMC13623573; doi:10.3389/fnut.2026.1888542)
Supplement: Supplementary file 6 [file Table_6.DOCX]

Supplementary table 6

| Model | AUC (95%CI) |
| --- | --- |
|  |  |
| In-hospital all-cause mortality |  |
| CONUT | 0.65 (0.58-0.72) |
| SOFA | 0.76 (0.70-0.83) |
| CONUT+SOFA | 0.78 (0.72-0.84) |
| Admission to the Intensive Care Unit |  |
| CONUT | 0.67 (0.63-0.70) |
| SOFA | 0.74 (0.71-0.77) |
| CONUT+SOFA | 0.76 (0.73-0.79) |
| Prolonged hospital stay>14days |  |
| CONUT | 0.61 (0.57-0.64) |
| SOFA | 0.56 (0.52-0.59) |
| CONUT+SOFA | 0.61 (0.58-0.65) |
