# Supplementary material for: Clinical significance of controlling nutritional status score in predicting ICU admission, prolonged hospitalization, and in-hospital mortality in emergency sepsis: a single-center retrospective study
Source: Front Nutr. 2026 Sep 16;13:1888542. doi: 10.3389/fnut.2026.1888542 (PMC13623573; doi:10.3389/fnut.2026.1888542)
Supplement: Supplementary file 7 [file Table_7.DOCX]

Supplementary table 7

|  | DeLong test | *P* | Categorical NRI(95% CI） | *P* | Continuous NRI(95% CI） | *P* | IDI(95% CI） | *P* |
| --- | --- | --- | --- | --- | --- | --- | --- | --- |
| In-hospital all-cause mortality | | | | | | | | |
| Model1VS Model2 | 2.388 | **0.017** | 0.108[ 0.014 - 0.202 ] | **0.025** | 0.571[ 0.292 - 0.851 ] | **<.001** | 0.040[0.018 -0.062 ] | **<.001** |
| Model1VS Model3 | 3.569 | **<.001** | 0.109[ 0.016 - 0.203 ] | **0.022** | 0.701[ 0.423 - 0.980 ] | **<.001** | 0.048[0.025 -0.071 ] | **<.001** |
| Model2VS Model3 | 1.388 | 0.165 | 0.003[-0.056 - 0.061 ] | 0.930 | 0.380[ 0.096 - 0.665 ] | **0.009** | 0.008[ -0.001 - 0.017 ] | 0.077 |
| Admission to the Intensive Care Unit | | | | | | | | |
| Model1VS Model2 | -3.432 | **<.001** | 0.287[ 0.210 - 0.364] | **<.001** | 0.333[ 0.216 - 0.449 ] | **<.001** | 0.087[ 0.062 - 0.113] | **<.001** |
| Model1VS Model3 | -6.646 | **<.001** | 0.403[ 0.336 - 0.470 ] | **<.001** | 0.660[ 0.548 - 0.771 ] | **<.001** | 0.119[ 0.098 - 0.139] | **<.001** |
| Model2VS Model3 | -2.726 | **0.006** | 0.097[ 0.043 - 0.151 ] | **<.001** | 0.434[ 0.318 - 0.549 ] | **<.001** | 0.032[ 0.021 - 0.042] | **<.001** |
| Prolonged hospital stay>14days | | | | | | | | |
| Model1VS Model2 | 2.106 | **0.035** | -0.065[ -0.105 - -0.025] | **0.001** | -0.334[ -0.460 - -0.207] | **<.001** | -0.018[ -0.027 - -0.009] | **<.001** |
| Model1VS Model3 | -1.679 | 0.093 | 0.028[ -0.002 - 0.057] | 0.069 | 0.140[ 0.014 - 0.266 ] | **0.030** | 0.001[ -0.001 - 0.003 ] | 0.472 |
| Model2VS Model3 | -2.674 | **0.008** | 0.093[ 0.050 - 0.136 ] | **<.001** | 0.388[ 0.262 - 0.513 ] | **<.001** | 0.019[ 0.011 - 0.027 ] | **<.001** |

Model1:CONUT

Model2:SOFA

Model3:CONUT+SOFA
