# Supplementary material for: Clinical significance of controlling nutritional status score in predicting ICU admission, prolonged hospitalization, and in-hospital mortality in emergency sepsis: a single-center retrospective study
Source: Front Nutr. 2026 Sep 16;13:1888542. doi: 10.3389/fnut.2026.1888542 (PMC13623573; doi:10.3389/fnut.2026.1888542)
Supplement: Supplementary file 8 [file Table_8.DOCX]

Supplementary table 8. the threshold value and net benefit.

|  | Threshold Value | Treat All | Net Benefit |
| --- | --- | --- | --- |
| admission to the intensive care unit | | | |
| CONUT | 0.64 | -0.782407407 | 0.0004629629629629671 |
| SOFA | 0.78 | -1.916666667 | 0.001212121212121213 |
| CONUT+SOFA | 0.86 | -3.583333333 | 0.010476190476190483 |
| prolonged hospital stay>14days | | | |
| CONUT | 0.33 | -0.094527363 | 0.0005099502487562052 |
| SOFA | 0.31 | -0.062801932 | 0.003526570048309169 |
| CONUT+SOFA | 0.34 | -0.111111111 | 0.0022727272727272527 |
| in-hospital all-cause mortality | | | |
| CONUT | 0.09 | -0.028388278 | 0.0006593406593406598 |
| SOFA | 0.11 | -0.051498127 | 0.0005617977528089898 |
| CONUT+SOFA | 0.09 | -0.028388278 | 0.0004945054945054948 |

ICU admission: CONUT 0-64%，SOFA 0-78%，both combined 0-86%

Prolonged hospitalization: CONUT 0-33%，SOFA 0-31%，both combined 0-34%

In-hospital mortality:CONUT 0-9%，SOFA 0-11%，both combined 0-9%
